# Arsenic Exposure Perturbs the Gut Microbiome and Its Metabolic Profile in Mice: An Integrated Metagenomics and Metabolomics Analysis

**DOI:** 10.1289/ehp.1307429

**Published:** 2014-01-10

**Authors:** Kun Lu, Ryan Phillip Abo, Katherine Ann Schlieper, Michelle E. Graffam, Stuart Levine, John S. Wishnok, James A. Swenberg, Steven R. Tannenbaum, James G. Fox

**Affiliations:** 1 Department of Biological Engineering,; 2 Department of Biology, and; 3 Division of Comparative Medicine, Massachusetts Institute of Technology, Cambridge, Massachusetts, USA; 4 Department of Environmental Sciences and Engineering, University of North Carolina at Chapel Hill, Chapel Hill, North Carolina, USA; 5 Department of Chemistry, Massachusetts Institute of Technology, Cambridge, Massachusetts, USA

## Abstract

Background: The human intestine is host to an enormously complex, diverse, and vast microbial community—the gut microbiota. The gut microbiome plays a profound role in metabolic processing, energy production, immune and cognitive development, epithelial homeostasis, and so forth. However, the composition and diversity of the gut microbiome can be readily affected by external factors, which raises the possibility that exposure to toxic environmental chemicals leads to gut microbiome alteration, or dysbiosis. Arsenic exposure affects large human populations worldwide and has been linked to a number of diseases, including cancer, diabetes, and cardiovascular disorders.

Objectives: We investigated the impact of arsenic exposure on the gut microbiome composition and its metabolic profiles.

Methods: We used an integrated approach combining 16S rRNA gene sequencing and mass spectrometry–based metabolomics profiling to examine the functional impact of arsenic exposure on the gut microbiome.

Results: 16S rRNA gene sequencing revealed that arsenic significantly perturbed the gut microbiome composition in C57BL/6 mice after exposure to 10 ppm arsenic for 4 weeks in drinking water. Moreover, metabolomics profiling revealed a concurrent effect, with a number of gut microflora–related metabolites being perturbed in multiple biological matrices.

Conclusions: Arsenic exposure not only alters the gut microbiome community at the abundance level but also substantially disturbs its metabolic profiles at the function level. These findings may provide novel insights regarding perturbations of the gut microbiome and its functions as a potential new mechanism by which arsenic exposure leads to or exacerbates human diseases.

Citation: Lu K, Abo RP, Schlieper KA, Graffam ME, Levine S, Wishnok JS, Swenberg JA, Tannenbaum SR, Fox JG. 2014. Arsenic exposure perturbs the gut microbiome and its metabolic profile in mice: an integrated metagenomics and metabolomics analysis. Environ Health Perspect 122:284–291; http://dx.doi.org/10.1289/ehp.1307429

## Introduction

The human body is host to 100 trillion gut microbes, approximately 10 times more than all human cells (Ley et al. 2006). Ley et al. (2006) estimated that the approximately 500–1,000 species residing in the human gut encode 100-fold more unique genes than the human genome. The gut microbiota has important functions in metabolic processing, energy production, immune cell development, food digestion, epithelial homeostasis, and so forth (Young et al. 2008). Mounting evidence indicates that dysregulated gut microflora contributes in a significant way to a variety of diseases, including diabetes, obesity, cardiovascular diseases, allergies, inflammatory bowel disease, and others (Ley et al. 2005; Qin et al. 2012; Wang et al. 2011). For example, obese individuals exhibit a remarkable reduction in the abundance of Bacteroidetes and a relative increase in Firmicutes compared with lean individuals (Turnbaugh et al. 2006). Likewise, a metagenome-wide association study revealed that beneficial butyrate-producing bacteria are less abundant and that opportunistic pathogens are more abundant in individuals with diabetes than in healthy individuals (Qin et al. 2012). The gut microbiome evolves through several transitions during the first years of life and thereafter remains relatively constant if no significant perturbations occur. However, the composition of the gut microbiome is highly diverse, and this diversity can be readily affected by external factors such as environment, diet, bacterial/viral infection, and antibiotics. This raises the possibility that exposure to toxic environmental chemicals leads to gut microbiome alteration (dysbiosis) as a mechanism by which environmental agents exert their detrimental effects on human health.

Arsenic exposure affects large human populations worldwide, with contamination of drinking water by geological sources of inorganic arsenic being the primary route of exposure. Hundreds of millions of people around the world, especially in South and East Asia, drink water with arsenic levels that far exceed the 10-μg/L guideline established or accepted by the World Health Organization and the U.S. Environmental Protection Agency (EPA) (Hughes et al. 2011). In the United States, as many as 25 million people are estimated to drink water with an arsenic level > 10 μg/L because private wells are not regulated by the U.S EPA and other agencies (Kozul et al. 2009). Arsenic exposure has been associated with a number of diseases such as skin, bladder, lung, and liver cancers and diabetes, as well as cardiovascular disorders (Hughes et al. 2011; Van de Wiele et al. 2010). More recently, arsenic exposure has been linked to an increased incidence of diabetes in animal models and human population studies (Paul et al. 2011). Numerous mechanisms have been proposed for arsenic-induced diseases, including interactions between arsenic and sulfur, oxidative stress, genotoxicity, altered DNA repair and signal transduction, cell proliferation, and epigenetics (Hou et al. 2012; Hughes et al. 2011; Ren et al. 2011; Smeester et al. 2011). Accumulating evidence indicates that perturbations of the gut microbiome and its influence on metabolic and physiological functions may play an important role in the development of human diseases. Given the essential role of the gut microbiome in a variety of aspects of human health coupled with the high toxicity of arsenic, there is a need to elucidate the effects of arsenic exposure on the gut microbiome and its functions. In particular, several seminal studies have reported interactions between the gut microbiome and environmental chemicals such as arsenic, mercury, polycyclic aromatic hydrocarbons, and polychlorinated biphenyls (Choi et al. 2013; Liebert et al. 1997; Pinyayev et al. 2011; Van de Wiele et al. 2005, 2010).

The gut microbiome has profound roles in modulating host metabolism. For example, nondigestible carbohydrates are degraded via fermentation by the gut bacteria to yield energy for microbial growth and microbial end products that act as energy substrates, inflammation modulators, and signaling molecules (Holmes et al. 2011). Therefore, the reach of the gut microbiome on host metabolism extends well beyond local effects in the gut to diverse remote organ systems, such as liver, brain, adipose, and muscle (Claus et al. 2011; Diaz et al. 2011). Accumulating evidence indicates that metabolic perturbations associated with changes in the gut microbiome composition are important risk factors for developing diseases (Jones et al. 2008; Wang et al. 2011). For example, gut microflora–generated trimethylamine *N*-oxide from dietary choline and carnitine has been strongly associated with atherosclerosis in animal models and clinic cohorts (Wang et al. 2011). Therefore, it is of particular interest to probe the gut microbiome–related metabolic changes associated with an arsenic-perturbed gut microbiota community. In this aspect, mass spectrometry–based metabolomics profiling is highly attractive because of its high sensitivity, ability to detect molecules with diverse structures, wide dynamic range, quantitative capability, and ease of interfacing with other separation techniques such as liquid chromatography (Lu et al. 2012).

In the present study, we applied an integrated approach combining 16S rRNA gene sequencing and liquid chromatography–mass spectrometry (LC-MS) metabolomics to analyze the effects of arsenic exposure on the gut microbiome and its metabolite profiles. Metagenomics sequencing revealed that arsenic exposure significantly perturbed the gut microbiome composition in C57BL/6 mice. Our nontargeted metabolomics profiling revealed a notable effect of arsenic exposure in these mice, with diverse perturbed metabolites being corelated with gut microbiome changes.

## Materials and Methods

*Animals and exposure*. Specific-pathogen-free C57BL/6 female mice (~ 6 weeks of age) were purchased from Jackson Laboratories (Bar Harbor, ME). Mice were provided pelleted rodent diet (ProLab 3000; Purina Mills, Gray Summit, MO) and filtered water *ad libitum* and were maintained in facilities accredited by the Association for Assessment and Accreditation of Laboratory Animal Care International. A total of 20 mice (10 mice/group, body weight = 20 ± 3 g) were housed in static microisolator cages (5 mice/cage) on heat-treated hardwood bedding, under environmental conditions of 22°C, 40–70% humidity, and a 12:12 hr light:dark cycle. All experiments were approved by the Massachusetts Institute of Technology Committee on Animal Care. The animals were treated humanely and with regard for the alleviation of suffering. Inorganic arsenic (arsenic, 10 ppm) was administered to mice (~ 8 weeks of age) as sodium arsenite (Fisher Scientific, Waltham, MA) in drinking water for 4 weeks. Freshly prepared arsenic-containing water (10 ppm) was provided to mice every Monday and Thursday. Control mice received water alone.

*Animal monitoring and histological analysis*. Throughout the experiments, mice were assessed daily for evidence of diarrhea, dehydration, and deteriorating body condition. Mice were euthanized with carbon dioxide and necropsied after 4 weeks of arsenic consumption. Formalin-fixed tissues were routinely processed, embedded in paraffin, sectioned at 4 μm, stained with hematoxylin and eosin, and evaluated by a board-certified veterinary pathologist blinded to the sample identity. Inflammation, edema, epithelial defects, hyperplasia, and dysplasia of multiple regions of liver (left lateral, medial, right lateral, and caudate lobes) and colon (distal, transverse, and proximal colon) were scored on an ascending scale (0–4, with 0 being normal) of severity and invasiveness of the lesion, if any. Pathological scores did not show any significant difference between the control and arsenic-treated mice and are not presented here. We also did not observe any significant changes in body weights, mortality, and food intake.

*16S rRNA gene sequencing*. We isolated DNA from fecal pellets collected during necropsy using a PowerSoil® DNA Isolation Kit (MO BIO Laboratories, Carlsbad, CA) according to the manufacturer’s instructions. The resultant DNA was quantified by ultraviolet spectroscopy and stored at –80°C for further analysis. DNA was amplified using universal primers of U515 (GTGCCAGCMGCCGCGGTAA) and E786 (GGACTACHVGGGTWTCTAAT) to target the V4 regions of 16S rRNA of bacteria. Individual samples were barcoded, pooled to construct the sequencing library, then sequenced using an Illumina Miseq (Illumina, San Diego, CA) to generate pair-ended 150 × 150 reads.

*Analysis of 16S rRNA sequencing data*. The raw mate-paired fastq files were quality-filtered, demultiplexed, and analyzed using Quantitative Insights into Microbial Ecology (QIIME) software (http://qiime.org). For quality filtering, the default parameters of QIIME were maintained in which reads with a minimum Phred quality score of < 20, containing ambiguous base calls and < 113 bp of consecutive high-quality base calls, were discarded. In addition, reads with three consecutive low-quality bases were truncated. The samples sequenced were demultiplexed using 8-bp barcodes, allowing 1.5 errors in the barcode. UCLUST software (http://www.drive5.com/uclust) was used to choose the operational taxonomic units (OTUs) with a threshold of 97% sequence similarity. A representative set of sequences from each OTU was selected for taxonomic identification of each OTU using the Ribosomal Database Project (RDP) classifier (http://rdp.cme.msu.edu). The Greengenes OTUs (4feb2011 build) reference sequences (97% sequence similarity) were used as the training sequences for RDP classifier. A 0.80 confidence threshold was used for taxonomic assignment. The taxonomic assignment of 16S rRNA sequencing data could be achieved at different levels, including phylum, class, order, family and genus. Our analyses were typically conducted at the family level because of the higher confidence in the assignment of taxa based on the sequencing reads; therefore, a significant change at the family level may reflect changes of multiple gut bacteria at genus and species levels. The sequencing data have been deposited in the MG-RAST (metagenomics Rapid Annotation using Subsystem Technology) server (http://metagenomics.anl.gov/).

*Sample processing for metabolomics*. One day before euthanasia of the mice, we collected urine samples using a metabolic cage with dry ice placed around the urine collection vessel to prevent oxidation or degradation of metabolites during the collection period (approximately 16 hr). Fecal pellets were also collected from individual animals. We collected plasma samples during necropsy. Metabolites were isolated from urine using methanol as described previously (Lu et al. 2012). Cold methanol (80 μL) was added to 20 μL urine or plasma. After vortexing for 1 min, the samples were incubated at 4°C for 20 min and then centrifuged for 10 min at 12,000 rpm. The supernatant was collected, dried in a SpeedVac (Savant SC110A; Thermo Electron, Waltham, MA), and then resuspended in 30 μL 98:2 water:acetonitrile for MS analysis. Metabolite extraction from fecal pellets was conducted in a similar manner. Fecal pellets (25 mg) were dissolved in 400 μL cold methanol solution (50:50 methanol:water), followed by vortexing at maximum speed for 10 min using a flat-bed vortex (MO BIO Laboratories). The supernatant was centrifuged for 10 min at 12,000 rpm, dried in a SpeedVac, and then resuspended in 30 μL 98:2 water:acetonitrile for metabolomics profiling.

*Metabolomics profiling*. We performed LC-MS analyses on a quadrupole-time-of-flight (Q-TOF) 6510 mass spectrometer (Agilent Technologies, Santa Clara, CA) with an electrospray ionization source. The mass spectrometer was interfaced with an Agilent 1200 HPLC system. The Q-TOF was calibrated daily using the standard tuning solution from Agilent Technologies. The typical mass accuracy of the Q-TOF was < 10 ppm. Metabolites were analyzed in the positive mode only over a range of 80–1000 *m/z* using a C18 T3 reverse-phase column from Waters Corporation (Milford, MA) because of the higher numbers of detected molecular features (i.e., metabolites), as demonstrated previously (Lu et al. 2012). Metabolomics profiling data were processed as described previously (Lu et al. 2012). MS/MS was generated on the Q-TOF to confirm the identity of perturbed metabolites. The metabolomics data were submitted to the XCMS Online server (https://xcmsonline.scripps.edu/).

*Data processing of metabolomics data*. Data acquired in Agilent .d format were converted to mzXML using MassHunter Workstation software from Agilent Technologies. Data were filtered by intensity, and only signals with intensities > 1,000 were considered. The converted data were processed using XCMS Online for peak picking, alignment, integration, and extraction of the peak intensities. To profile individual metabolite differences between control and arsenic-treatment groups, a two-tailed Welch’s *t*-test was used (*p* < 0.05). The exact masses of molecular features with significant changes were searched against the Human Metabolome Database (HMDB; http://www.hmdb.ca/), METLIN (http://metlin.scripps.edu), and Kyoto Encyclopedia of Genes and Genomes (KEGG) databases (http://www.genome.jp/kegg/pathway.html) with a 10-ppm mass accuracy threshold. The matched exact masses were stored and used for the generation of MS/MS data to identify the metabolites.

*Statistical analysis of data*. Principal component analysis (PCA) was performed to examine intrinsic clusters of metabolomics data. A 95% confidence interval (CI) was used as the threshold to identify potential outliers in all samples. In addition, heat maps were generated using a hierarchical clustering algorithm to visualize the metabolite difference within the data set. Principal coordinate analysis (PCoA) was used to compare the gut microbiome profiles between the control and treatment. The difference in the gut microbiome composition was further assessed using a nonparametric test via Metastats software (http://metastats.cbcb.umd.edu/) as described previously (White et al. 2009). The correlation matrix between the gut microflora–related metabolites and gut bacterial species was generated using Pearson’s corelation coefficient.

## Results

*Workflow to probe functional changes of the gut microbiome*. The experimental workflow combined 16S rRNA gene sequencing and metabolite profiling to examine the impact of arsenic exposure on the gut microbiome and its metabolic profiles (see Supplemental Material, Figure S1). Briefly, DNA was isolated from fecal pellets, amplified by polymerase chain reaction using 16S rRNA–specific primers, followed by 150 × 150 bp paired-end sequencing using the Illumina Miseq platform. The resultant sequencing reads were processed using the QIIME and Metastats software packages to reveal exposure-induced gut microbiome changes. For metabolomics analysis, metabolites from fecal pellets, urine, and plasma were extracted and analyzed by Q-TOF. Molecular features (i.e., all signals associated with a given analyte) were further processed and statistically analyzed with XCMS software to profile metabolites with significant changes (1.5-fold change, *p* < 0.05) between the control and arsenic-treated animals. The resultant peak list with exact masses was searched against metabolite databases, including HMDB and METLIN. Next, the matched exact masses and associated retention times were used to generate MS/MS spectra to confirm the metabolite identities, followed by metabolic pathway or function analysis with the KEGG and HMDB databases. Finally, correlations between the gut microbiome changes and shifted metabolome were examined to establish the functional impacts of arsenic exposure on the gut microbiome.

**Figure 1:**
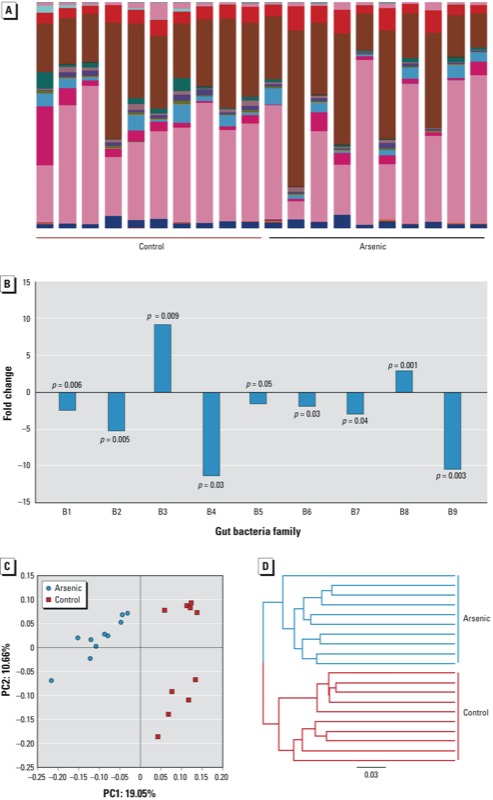
(*A*) The gut microbiome composition profiles at the family level in the control and arsenic-treated mice revealed by 16S rRNA sequencing (each color represents one bacterial family). (*B*) The fold changes and taxa assignments of significantly perturbed gut bacteria in arsenic-treated mice compared with controls [Abbreviations: c, class; f, family; o, order; p, phylum; and family abbreviations: B1 (Other;Other;Other;Other); B2 (p_Cyanobacteria;c_Chloroplast;o_Streptophyta;f_unassigned); B3 (p_Firmicutes;c_Bacilli;o_Bacillales;Other); B4 (p_Firmicutes;c_Clostridia;Other;Other); B5 (p_Firmicutes;c_Clostridia;o_Clostridiales;f_unassigned); B6 (p_Firmicutes;c_Clostridia;o_Clostridiales;f_Catabacteriaceae); B7 (p_Firmicutes;c_Clostridia;o_Clostridiales;f_Clostridiaceae); B8 (p_Firmicutes;c_Clostridia;o_Clostridiales;f_Clostridiales Family XIII Incertae Sedis); B9 (p_Tenericutes;c_Erysipelotrichi;o_Erysipelotrichales;f_Erysipelotrichaceae).] (*C*) The gut microbiome patterns of control and arsenic-treated mice differentiated by principal coordinate analysis. (*D*) Hierarchical clustering analysis by UPGMA indicates that controls and arsenic-treated mice clustered in their own groups, with the UPGMA distance tree constructed at a distance of 0.03.

*Arsenic-induced gut microbiome changes*. Figure 1A shows the identified gut bacteria assigned at the family level from 16S rRNA sequencing reads, with each color representing an individual bacterial family (see also Supplemental Material, Figure S2A and Table S1). In terms of the assignment at the phylum level, Firmicutes (52.79%) and Bacteroidetes (41.57%) were predominant in the gut bacteria of mice, followed by Tenericutes (3%), Actinobacteria (0.18%), Cyanobacteria (0.023%), and Proteobacteria (0.0042%), with 2.41% sequences unmatched with the database (see Supplemental Material, Figure S2B). Our observations and assignments of gut bacteria at phylum level are consistent with previous reports that the gut microbiome consists of only several phyla (Turnbaugh et al. 2006). The taxonomic assignments and fold changes of gut bacterial components that were significantly changed (*p* < 0.05) are listed in Figure 1B. The difference in the gut microbiome patterns arising from arsenic is readily differentiated using multivariate statistical analysis, as shown by the PCoA plot in Figure 1C. The control and treated animals are well separated, with 19.95% and 10.66% variation explained by principal component (PC) 1 and PC2, respectively. Consistent with the PCoA plot, the jackknifed beta diversity and hierarchical clustering analysis via the unweighted pair group method with arithmetic mean (UPGMA) demonstrated that all control and treated animals clustered in their own groups, as shown in Figure 1D. In addition, control mice clustered into two subgroups in the PCoA plot (Figure 1C) and UPGMA hierarchical clustering analysis (Figure 1D), which was attributed to individual variations of gut microbiome profiles, as illustrated in Figure 1A.

**FIgure 2:**
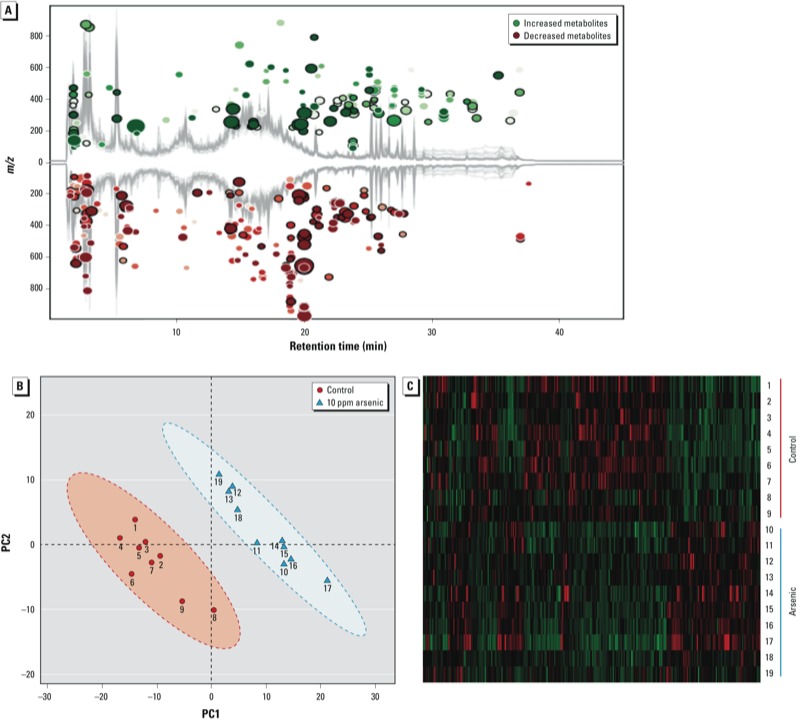
(*A*) Arsenic exposure perturbed the metabolic profile of fecal samples of mice, with 370 molecular features being significantly changed compared with controls (fold change > 1.5 and p < 0.05). (*B*) Controls were separated from arsenic-treated mice in metabolite profiles by PCA. (*C*) Hierarchical clustering heat map constructed using molecular features with 1.5-fold changes (*p* < 0.05) shows a consistent clustering pattern within individual groups.

*Arsenic-induced changes in metabolic profiles of the gut microbiome*. The combination in feces of a large quantity of gut bacteria and their metabolic products creates an ideal biological sample to assess functional changes of the gut microbiome. Figure 2A illustrates that arsenic exposure perturbed the metabolic profiles of the gut microbiome, with 146 increased and 224 decreased molecular features, respectively. As shown in Figure 2B, the control and arsenic-treated groups could be differentiated readily using metabolite fingerprints, with an excellent separation of the control and arsenic-treated animals using the first two components of PCA (i.e., PC1 and PC2). The hierarchical clustering heat map in Figure 2C also shows similar clustering patterns of detected molecular features within each group. Clear separations of metabolite profiles between control and arsenic-treated mice were also observed for the urine and plasma samples, with a large number of perturbed molecular features (see Supplemental Material, Figure S3). A number of metabolites with > 1.5-fold changes between the control and arsenic-treated mice were identified (see Supplemental Material, Tables S2–S4) using the MS/MS approach (see Supplemental Material, Figure S4). The structures of these metabolites were diverse, including amino acid derivatives, bile acids, lipids, fatty acids, isoflavones, indole derivatives, and glucuronide and carnitine conjugates, with many of the metabolites being either directly generated or modulated by the gut bacteria.

**Figure 3:**
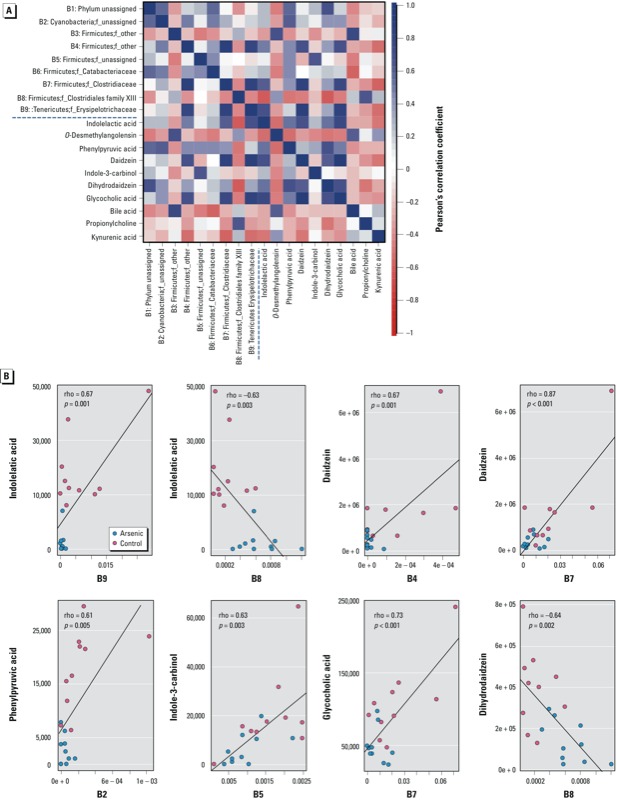
(*A*) Correlation plot showing the functional correlation between perturbed gut bacteria families and altered fecal metabolites. (*B*) Scatter plots illustrating statistical association between the relative abundance of altered gut bacteria families and the mass spectrum intensities of some typical gut microflora–related metabolites, including indole-containing compounds, isofavone metabolites, and bile acids.

*Correlation between the gut microbiome and metabolites*. To explore the functional correlation between the gut microbiome changes and metabolite perturbations, a correlation matrix was generated by calculating the Pearson’s correlation coefficient (Figure 3A). Clear correlations could be identified between the perturbed gut microbiome and altered metabolite profiles (*p* > 0.5 or < –0.5, *p* < 0.05). Figure 3B lists several typical gut microflora–related metabolites that are highly correlated with specific gut bacteria to demonstrate the functional correlation between the gut microbiome and metabolites. For example, indolelactic acid, which decreased 11.6-fold in arsenic-treated mice, positively correlates with the B9 family (p_Tenericutes;f_Erysipelotrichaceae), but negatively correlates with the B8 family (p_Firmicutes;f_Clostridiales Family XIII Incertae Sedis). Likewise, daidzein positively correlates with the B4 (p_Firmicutes;f_Other) and B7 (p_Firmicutes;f_Clostridiaceae) families, respectively. Phenylpyruvic acid, indole-3-carbinol, and glycocholic acid positively correlate with the B2 (p_Cyanobacteria;f_unassigned), B5 (p_Firmicutes;f_unassigned), and B7 (p_Firmicutes;f_Clostridiaceae) families, respectively, whereas dihydrodaidzein negatively correlates with the B8 family (p_Firmicutes;f_Clostridiales Family XIII Incertae Sedis). In summary, arsenic exposure induces a significant taxonomic perturbation in the gut microbiome, which in turn substantially alters the metabolomic profile of the gut microbiome, as evidenced by changes of diverse gut microflora–related metabolites.

## Discussion

We used high-throughput 16S rRNA gene sequencing and metabolomics profiling to study the impact of arsenic exposure on the gut microbiome and its metabolic profiles. The data clearly show that arsenic exposure induced a significant change in the gut microbiome composition of mice. In addition, these perturbed gut bacteria were strongly associated with changes of a number of gut microflora–related metabolites, indicating that arsenic exposure not only disturbs gut bacteria at the abundance level but also substantially alters the metabolomic profile of the gut microbiome, resulting in the disturbance of host metabolite homeostasis after arsenic exposure. These findings may provide mechanistic insights regarding perturbations of the gut microbiome as a new mechanism of environmental chemical–induced human disease.

Accumulating evidence suggests that metabolic changes associated with gut microbiome perturbations are important risk factors for inducing abnormal tissue functions resulting in diseases such as obesity, insulin resistance, and cardiovascular disease (Jones et al. 2008; Turnbaugh et al. 2006; Wang et al. 2011). The gut microbiome could directly change its metabolic capacity and affect intestinal function locally through microbial products. For example, the gut microbiota has a strong effect on energy homeostasis in the colon, attributed to the use of butyrate produced by gut bacteria as the primary energy source for colonocytes (Donohoe et al. 2011). Likewise, altered gut microbiota can also trigger systemic effects on host metabolism in remote tissues such as liver, brain, adipose, and muscle because the products of microbial metabolism could serve as signaling molecules or act in conjunction with the host on the metabolism of diverse chemicals to affect individual susceptibility to different diseases. For instance, trimethylamine *N*-oxide, a gut microflora–generated metabolite, has been identified to be strongly associated with atherosclerosis in a large clinical cohort (Wang et al. 2011). Dietary choline–enhanced atherosclerosis could be inhibited by suppressing intestinal microflora in atherosclerosis-prone mice, further highlighting the role of perturbations of gut microflora–generated metabolites in disease development.

The gut microbiome plays a key role in the energy metabolism of the host. Nondigestible carbohydrates are degraded via fermentation in the colon by the gut microbiota to yield energy for the host and metabolic end products such as short-chain fatty acids. Previous studies have documented that the imbalanced gut microbiome may be associated with human diseases such as obesity and diabetes (Qin et al. 2012; Turnbaugh et al. 2006). For example, Turnbaugh et al. (2006) found that obesity was associated with a large shift in the relative abundance of the specific taxa present, with a statistically significant reduction in Bacteroidetes and a significantly greater proportion of Firmicutes in obese mice (Turnbaugh et al. 2006). Likewise, transplantation of the cecal microbiota from obese mice fed on high-fat diets into germ-free recipients increases adiposity significantly more than transplantation of microbiota from lean mice (Turnbaugh et al. 2006), further highlighting the role of the gut microbiome in affecting the efficiency of harvesting energy and defining obese/lean phenotypes. Cho et al. (2012) also demonstrated the influence of gut microbiome perturbations on tissue adiposity of the host by delineating the effect of antibiotics use in early life. These authors discovered substantial taxonomic changes in the gut microbiome, changes in primary genes responsible for the metabolism of carbohydrates to short-chain fatty acids, and marked alterations in hepatic metabolism of lipids and cholesterol. In the present study, bacteria in the family of Bacteroidetes were not increased by arsenic exposure, whereas four Firmicutes families were significantly decreased. Arsenic is not an obesogen, but rather has anti-obesogenic properties, although the mechanism remains unknown (Paul et al. 2011). Excess accumulation of white adipose tissue in obesity is a risk factor for insulin resistance and the development of diabetes because white adipose tissue regulates energy balance, lipid, and glucose homeostasis, and release a variety of signaling factors that affect insulin sensitivity and inflammation (Rosen and Spiegelman 2006). However, defects in adipogenesis in adipose tissue can also lead to the development of insulin resistance and diabetes (Hou et al. 2012; Vigouroux et al. 2011). We identified several decreased Firmicutes families following arsenic exposure, and a number of Firmicutes species, such as *Eubacterium, Faecalibacterium,* and *Roseburia,* are known to be butyrate producers (Tremaroli and Backhed 2012). Thus, an arsenic-altered gut microbiome may affect energy harvesting, short-chain fatty acid production, and adipogenesis. Another example to support arsenic exposure impairment of energy metabolism stems from the observation that fatty acid carnitines were significantly reduced in the urine of arsenic-treated mice (see Supplemental Material, Table S3). Fatty acid carnitines are used to transport fatty acids into mitochondria for fatty acid oxidation to generate metabolic energy; this process may be used to compensate for insufficient energy harvest due to disturbed gut microbiota after arsenic exposure. In agreement with this potential mechanism, germ-free mice and lean individuals also exhibit increased fatty acid oxidation and decreased lipogenesis (Backhed et al. 2007).

Indole-containing metabolites were significantly altered in fecal and urine samples after arsenic exposure in mice (see Supplemental Material, Figure S5A). The presence of the altered indole-containing metabolites is highly correlated with perturbed gut bacterial families. For example, 3-indolepropionic acid may serve as a specific indicator of imbalanced gut bacteria because a gut bacterial metabolic process is needed to synthesize this compound. A recent study identified *Clostridium sporogenes* as the only species, among 24 intestinal microflora tested, to produce 3-indolepropionic acid (Wikoff et al. 2009). Thus, increased excretion of 3-indolepropionic acid in urine (+1.8-fold) may indicate that the abundance of *Clostridium sporogenes* or species with similar functions have increased. In fact, among six significantly perturbed Firmicutes families, one Firmicutes family significantly increased in abundance after arsenic exposure—p_Firmicutes;c_Clostridia;o_Clostridiales;f_Clostridiales Family XIII Incertae Sedis, B8 in Figure 1B, with a +2.0-fold change. In particular, *Clostridium sporogenes* and this increased gut bacteria family are classified into the same order of Clostridiales. The formation of indole-containing metabolites likely occurs via the generation of indole by the intestinal bacteria that produce the enzymes to catalyze the conversion of tryptophan to indole, followed by further enzymatic processing. For example, absorbed indole could be converted to indoxyl in the liver, and then sulfated to allow for urinary excretion. An increased amount of indoxyl in urine (+2.9-fold in arsenic-treated mice) suggests an increased indole production by gut bacteria, which also supports the production of increased amounts of other urine indole-related metabolites such as IPA, indole-3-carboxylic acid, and indoleacrylic acid. However, increased urinary excretion of indoxyl may also suggest that phase I/II biotransformations—including hydroxylation, sulfation, and glucuronidation—were changed during exposure. In an elegant study, Wikoff et al. (2009) demonstrated a broad phase II metabolic response of the host to metabolites generated by the microbiome, as evidenced by the exclusive presence of numerous sulfated, glycine-conjugated, and glucuronide adducts in the serum of mice with normal gut microbiota compared with germ-free mice. In the present study, we also found several glucuronide metabolites to be significantly reduced in urine (see Supplemental Material, Table S3), possibly reflecting an impaired glucuronidation capacity affected by a perturbed gut microbiome as a result of arsenic exposure.

Isoflavone metabolites are also sensitive to gut microbiome changes (see Supplemental Material, Figure S5B). Previously, we discovered that daidzein was significantly increased in the serum of Rag2^–/–^ mice infected with *Helicobacter hepaticus* (Lu et al. 2012). The increased amount of daidzein was attributed to an altered gut microflora induced by *Helicobacter hepaticus* infection consistent with previous studies that demonstrated that *Helicobacter* spp. infections perturb the gut microflora in mice (Whary et al. 2006). Daidzein is converted by anaerobic bacteria in the large intestine to several metabolites—including dihydrodaidzein, *O*-desmethylangolensin, and equol—depending on the gut bacterial composition of the host. In the present study, we detected significantly decreased daidzein and dihydrodaidzein but increased *O*-desmethylangolensin in feces, suggesting that the formation of *O*-desmethylangolensin is favored in arsenic-treated mice. Of particular interest, *O*-desmethylangolensin is positively correlated with a Firmicutes family (p_Firmicutes; c_Bacilli;o_Bacillales;Other, +9.2-fold in arsenic-treated mice). Hur et al. (2000) suggested that several candidate bacteria are participating in daidzein metabolism, although the intestinal bacteria responsible for daidzein metabolism in humans have not been unambiguously identiﬁed. Nevertheless, altered isoflavone metabolites may be used as bioindicators to probe changes in the gut microbiome composition arising from diverse environmental factors such as bacterial infections (Lu et al. 2012) and toxic chemicals.

Bile acids and intermediates were significantly perturbed in arsenic-treated mice (see Supplemental Material, Table S2). Bile acids are cholesterol derivatives synthesized in the liver, and they undergo extensive enterohepatic recycling and gut microbial modification, including deconjugation and dehydroxylation. Bile acids not only have important functions in solubilizing cholesterol and facilitating the absorption of cholesterol, fat-soluble vitamins, and lipids from the gut, but they also act as signaling molecules to regulate metabolic homeostasis by activating diverse nuclear receptors (Nguyen and Bouscarel 2008). In the present study, 7-α-hydroxy-3-oxo-4-cholestenoate and its degradation product were excreted at a higher level in arsenic-treated mice compared with controls. 7-α-hydroxy-3-oxo-4-cholestenoate is involved in the biosynthesis of primary bile acids (Kanehisa et al. 2012), and an increased excretion of 7-α-hydroxy-3-oxo-4-cholestenoate and its degradation product may impair the synthesis and enterohepatic recycling of bile acids. Likewise, levels of glycocholic acid decreased (–2.3-fold) in fecal samples of arsenic-treated mice. These altered bile acid species indicate that arsenic exposure affects the homeostasis of bile acids. The underlying mechanisms remain elusive; however, arsenic-induced gut microbiome perturbations may play a role in this process. Previous studies demonstrated a large effect of the gut microflora on primary and secondary bile acid profiles in tissues of antibiotic-treated rats (e.g., Swann et al. 2011). Moreover, specific microbial bile acid co-metabolites present in peripheral tissues and pathway changes regulated by farnesoid X receptor indicate a broad signaling role for bile acids and highlight the symbiotic microbial influences in bile acid homeostasis in the host (Swann et al. 2011). Moreover, the role of gut bacteria in the regulation of bile acids has raised the question of whether bile acids modulated by the gut microbiome are associated with regulation of the host immune system (Brestoff and Artis 2013). Previous studies have demonstrated that bile acid signaling via their receptors is linked to a common antiinflammatory response in macrophages and monocytes (Brestoff and Artis 2013). In particular, it has been reported that chronic arsenic exposure significantly compromises the immune response to influenza A infection (Kozul et al. 2009). Clearly, further research is needed to shed light on the role of the gut microbiome and its associated metabolites, including bile acids, in arsenic-induced impaired immune responses and other diseases.

In the present study, we demonstrated that arsenic exposure altered the gut microbiome and associated metabolomic profiles. However, future studies are warranted to delineate the mechanistic basis of these perturbations. As shown by the correlation analysis between the gut bacteria and metabolites, arsenic exposure can induce changes in the gut microbiome in terms of abundance, resulting in a shifted metabolome in the gut microbiome. Of equal importance, arsenic exposure may also cause an altered metabolome by affecting the physiology of the gut bacteria without changing the species and abundance. Therefore, the changes in the metabolic profiles of the gut microbiome may not entirely depend on shifts in the spectrum of microbes revealed by 16S rRNA gene sequencing. Such metabolic changes could be achieved via other mechanisms besides altering the types and numbers of bacteria present in the gut, such as the regulation of gene and protein expression in a bacterium. Maurice et al. (2013) have recently demonstrated that xenobiotics significantly altered the physiology and gene expression of the human gut microbiome. Therefore, metatranscriptomics and metaproteomics profiling are warranted in the future to elucidate the role of arsenic exposure in altering the functions of the gut microbiome, which encodes 100-fold more unique genes than the human genome and profoundly modulates host metabolism (Ley et al. 2006).

Our findings show that arsenic exposure perturbs the gut microbiome composition and associated metabolic profiles in mice and represents an initial and critical step toward understanding how arsenic exposure affects the gut microbiome and its functions. Future studies are needed to address many intriguing questions in this exciting field. For example, it remains to be determined whether any changes to the gut microbiome and its associated metabolites occur in arsenic-exposed humans because there are differences in the gut microbiome between mice and humans. The use of epidemiological studies combined with experiments in humanized gnotobiotic mice will allow researchers to better address species differences and elucidate the interaction between arsenic exposure and the gut microbiome in humans. In addition, whereas we treated mice with 10 ppm arsenic for 4 weeks, humans are usually exposed to much lower doses of arsenic for longer periods. Thus, the dose- and time-dependent effects of arsenic exposure on the gut microbiome also need to be defined. Of particular importance is how arsenic impacts the gut microbiome during the windows of susceptibility because the establishment of the gut microflora is a temporal process occurring after birth (Palmer et al. 2007). Thus, arsenic-induced perturbations in the gut microbiome may have age-related effects. Likewise, answers to other issues, such as sex-specific influences, persistent effects of arsenic on the microbiome after cessation of exposure, and correlations between gut microbiome changes and toxicity phenotypes, all await future studies.

## Conclusions

We combined 16S rRNA sequencing and metabolomics to analyze the impact of arsenic on the gut microbiome and its metabolic profiles in mice. The sequencing revealed that arsenic exposure significantly altered the gut microflora composition, whereas the metabolomics experiments showed that a number of metabolites involved in diverse metabolic pathways were substantially perturbed after exposure to arsenic. In addition, correlation analysis identified that some gut bacteria families were highly correlated with altered gut microflora–related metabolites. Taken together, these data indicate that arsenic exposure not only perturbs the gut microbiome at the abundance level but that it also alters metabolic profiles of the gut microbiome, supporting the hypothesis that perturbations of the gut microbiome may serve as a new mechanism by which arsenic exposure leads to or exacerbates human diseases. Furthermore, these modulated gut microflora–related metabolites may be potential biomarkers useful for probing the functional impacts of arsenic and other diverse environmental chemicals on the gut microbiome.

## Supplemental Material

(2.5 MB) PDF
