# Supplementary material for: Arsenic Exposure Perturbs the Gut Microbiome and Its Metabolic Profile in Mice: An Integrated Metagenomics and Metabolomics Analysis
Source: Environ Health Perspect. 2014 Jan 10;122(3):284–91. doi: 10.1289/ehp.1307429 (PMC3948040; doi:10.1289/ehp.1307429)
Supplement: (2.5 MB) PDF [file ehp.1307429.s001.pdf]

Supplemental Material

Arsenic Exposure Perturbs the Gut Microbiome and Its Metabolic Profile in Mice: An Integrated Metagenomics and Metabolomics Analysis

Kun Lu, Ryan Phillip Abo, Katherine Ann Schlieper, Michelle E. Graffam, Stuart Levine, John S. Wishnok, James A. Swenberg, Steven R. Tannenbaum, and James G. Fox

Table of Contents

Supplemental Material, Table S1 p. 2

Supplemental Material, Table S2 p. 3

Supplemental Material, Table S3 p. 4

Supplemental Material, Table S4 p. 6

Supplemental Material, Figure S1 p. 7

Supplemental Material, Figure S2 p. 8

Supplemental Material, Figure S3 p. 9

Supplemental Material, Figure S4 p. 10

Supplemental Material, Figure S5 p. 11

Supplemental Material, Table S1. Relative abundance of typical gut bacterial families in the measurements for control and arsenic exposed mice.[a]

Taxa	Relative abundance (control)	Standard error [b] (control)	Relative abundance (arsenic)	Standard error (arsenic)
Root;k__Bacteria;Other;Other;Other;Other	3.67E-02	3.66E-03	3.37E-02	4.37E-03
Root;k__Bacteria;p__Actinobacteria;c__Actinobacteria;o__Bifidobacteriales;f__Bifidobacteriaceae	8.39E-04	4.09E-04	2.11E-03	8.85E-04
Root;k__Bacteria;p__Actinobacteria;c__Actinobacteria;o__Coriobacteriales;f__	0.00E+00	0.00E+00	8.29E-06	4.76E-06
Root;k__Bacteria;p__Actinobacteria;c__Actinobacteria;o__Coriobacteriales;f__Coriobacteriaceae	1.03E-03	3.45E-04	1.40E-03	5.26E-04
Root;k__Bacteria;p__Bacteroidetes;c__Bacteroidia;o__Bacteroidales;f__	4.16E-01	3.65E-02	4.46E-01	6.85E-02
Root;k__Bacteria;p__Bacteroidetes;c__Bacteroidia;o__Bacteroidales;Other	3.18E-05	1.36E-05	6.60E-05	1.64E-05
Root;k__Bacteria;p__Bacteroidetes;Other;Other;Other	1.52E-05	8.09E-06	1.87E-06	1.87E-06
Root;k__Bacteria;p__Cyanobacteria;c__Chloroplast;o__Streptophyta;f__	2.40E-04	9.37E-05	4.49E-05	1.66E-05
Root;k__Bacteria;p__Firmicutes;c__Bacilli;o__Bacillales;f__Bacillaceae	0.00E+00	0.00E+00	5.96E-06	4.31E-06
Root;k__Bacteria;p__Firmicutes;c__Bacilli;o__Bacillales;f__Staphylococcaceae	3.05E-06	3.05E-06	0.00E+00	0.0E+00
Root;k__Bacteria;p__Firmicutes;c__Bacilli;o__Bacillales;Other	1.96E-05	8.12E-06	1.80E-04	7.05E-05
Root;k__Bacteria;p__Firmicutes;c__Bacilli;o__Lactobacillales;f__Enterococcaceae	3.92E-06	3.92E-06	1.35E-05	6.95E-06
Root;k__Bacteria;p__Firmicutes;c__Bacilli;o__Lactobacillales;f__Lactobacillaceae	5.67E-02	2.31E-02	3.14E-02	8.48E-03
Root;k__Bacteria;p__Firmicutes;c__Bacilli;o__Lactobacillales;Other	1.23E-05	6.49E-06	0.00E+00	0.0E+00
Root;k__Bacteria;p__Firmicutes;c__Bacilli;o__Turicibacterales;f__Turicibacteraceae	3.48E-02	9.00E-03	3.14E-02	7.60E-03
Root;k__Bacteria;p__Firmicutes;c__Bacilli;Other;Other	1.48E-04	5.62E-05	9.60E-05	2.65E-05
Root;k__Bacteria;p__Firmicutes;c__Clostridia;o__Clostridiales;f__	1.62E-02	2.46E-03	9.81E-03	1.74E-03
Root;k__Bacteria;p__Firmicutes;c__Clostridia;o__Clostridiales;f__Catabacteriaceae	1.43E-02	2.53E-03	7.35E-03	1.85E-03
Root;k__Bacteria;p__Firmicutes;c__Clostridia;o__Clostridiales;f__Clostridiaceae	2.40E-02	7.19E-03	7.99E-03	2.21E-03
Root;k__Bacteria;p__Firmicutes;c__Clostridia;o__Clostridiales;f__Clostridiales Family XIII Incertae Sedis	2.26E-04	5.91E-05	6.59E-04	8.44E-05
Root;k__Bacteria;p__Firmicutes;c__Clostridia;o__Clostridiales;f__Dehalobacteriaceae	7.04E-04	7.40E-05	8.82E-04	1.25E-04
Root;k__Bacteria;p__Firmicutes;c__Clostridia;o__Clostridiales;f__Lachnospiraceae	2.97E-01	2.88E-02	3.28E-01	5.40E-02
Root;k__Bacteria;p__Firmicutes;c__Clostridia;o__Clostridiales;f__Ruminococcaceae	5.71E-02	4.00E-03	6.77E-02	9.20E-03
Root;k__Bacteria;p__Firmicutes;c__Clostridia;o__Clostridiales;Other	1.00E-02	1.68E-03	1.09E-02	2.89E-03
Root;k__Bacteria;p__Firmicutes;c__Clostridia;Other;Other	1.44E-04	5.67E-05	1.28E-05	8.89E-06
Root;k__Bacteria;p__Firmicutes;Other;Other;Other	9.27E-05	3.21E-05	5.33E-05	2.86E-05
Root;k__Bacteria;p__Proteobacteria;c__Gammaproteobacteria;o__Oceanospirillales;f__Halo monadaceae	7.44E-06	4.97E-06	1.02E-05	6.09E-06
Root;k__Bacteria;p__Proteobacteria;c__Gammaproteobacteria;o__Pseudomonadales;f__Morax ellaceae	1.48E-05	1.18E-05	1.20E-05	5.95E-06
Root;k__Bacteria;p__Tenericutes;c__Erysipelotrichi;o__Erysipelotrichales;f__Erysipelotrichaceae	6.86E-03	2.80E-03	6.54E-04	1.42E-04
Root;k__Bacteria;p__Tenericutes;c__Mollicutes;o__Anaeroplasmatales;f__Anaeroplasmataceae	2.08E-02	6.16E-03	1.62E-02	3.42E-03
Root;k__Bacteria;p__Tenericutes;c__Mollicutes;o__RF39;f__	2.39E-03	3.80E-04	1.88E-03	5.76E-04
Root;k__Bacteria;p__Tenericutes;Other;Other;Other	3.92E-06	3.92E-06	9.73E-07	9.73E-07
Root;Other;Other;Other;Other;Other	4.33E-03	9.14E-04	1.83E-03	2.76E-04

[a]The "Other" assignments are typically due to ambiguity when the RDP classifier tries to assign at the desired level. The “f_” or “f_unassigned” means that it matches a reference sequence, but that reference sequence is not well defined. [b]The mean and standard errors were calculated using the statistical tool of Metastats.

Supplemental Material, Table S2. Identified significantly changed metabolites (fold change >1.5 p < 0.05 compared to controls) by MS/MS in fecal samples.

Name	HMDB ID[a]	Fold	*p* value	Mass	Retention time	Gut flora related	Pathways or functions
O-Desmethylangolensin	HMDB04629	10.3	0.001	259.0971	22	Y	Isoflavone
Hydroxyphenylacetic acid	HMDB00440	3.6	0.05	153.054	15.1	Y	Tyrosine metabolsim
1b-Hydroxycholic acid or Tetrahydroxy-5b-cholanoic acid	HMDB00307	2.5	0.04	425.2936	23.7	Y	Bile acids
MG(0:0/18:2(9Z,12Z)/0:0)	HMDB11538	2.4	0.03	355.2822	33.1		Lipids
Cinnamaldehyde	HMDB03441	2.3	0.01	133.0647	23.8		Phenylpropanoid
7 alpha-Hydroxy-3-oxo-4-cholestenoate	HMDB12458	2.2	0.05	431.3119	29.6	Y	Bile acids
1b-Hydroxycholic acid	HMDB00307	2.1	0.05	425.2906	24.8	Y	Bile acids
Benzaldehyde	HMDB06115	2.0	0.04	107.0488	15.1	Y	
5-Phenylvaleric acid	HMDB02043	1.9	0.008	179.1058	19.9	Y	Fatty acids
Dimethylbenzoic acid	HMDB02237	1.8	0.05	151.074	15.3		Benzoic acid
Propionylcholine	HMDB13305	1.8	0.04	161.1416	2	Y	
Alpha-Linolenic acid	HMDB01388	1.7	0.04	279.2308	30.9		Fatty acids
3-Oxo-4,6-choladienoic acid	HMDB00476	1.7	0.05	371.258	26.1	Y	Bile acids
Kynurenic acid	HMDB00715	1.7	0.03	190.0489	21.1		Tryptophan metabolism
N-Acetylhistamine	HMDB13253	1.5	0.02	154.0969	1.9	Y	Amino acids and derivatives
2-Aminooctanoic acid	HMDB00991	1.5	0.04	160.1321	2.6		
Cytidine	HMDB00089	-1.6	0.02	244.0927	3.3		Nucleotide
N-a-Acetyl-L-arginine	HMDB04620	-1.8	0.005	217.1284	2		Amino acids and derivatives
Glycocholic acid	HMDB00138	-2.3	0.007	466.3143	25.8	Y	Bile acids
Guanosine	HMDB00133	-2.3	0.05	284.0966	3.1		Nucleoside
N6-Methyllysine	HMDB02038	-2.4	0.03	161.1276	4.4		Amino acids and derivatives
Dihydrodaidzein	HMDB05760	-3.0	0.002	257.0809	22.8	Y	Isoflavone
Cuminaldehyde	HMDB02214	-3.0	0.02	149.0947	18.3	Y	
Indole-3-carbinol	HMDB05785	-3.1	0.04	148.074	20.2	Y	Indoles
Daidzein	HMDB03312	-4.9	0.05	255.065	22.4	Y	Isoflavone
Phenylpyruvic acid	HMDB00205	-6.7	<0.0001	165.0539	14.9	Y	Phenylalanine metabolism
Indolelactic acid	HMDB00671	-11.6	0.0001	206.081	19.6	Y	Indoles

[a]The metabolites were indexed using the IDs in the Human Metabolome Database (HMDB, http://www.hmdb.ca/).

Supplemental Material, Table S3. Identified significantly changed metabolites (fold change >1.5 p < 0.05, compared to controls) in urine samples.

Name	HMDB ID[a]	Fold	*p* value	Mass	Retention time	Gut flora related	Pathways or functions
Dihydrogenistein	HMDB05897	3.8	0.04	273.07639	23.3	Y	Isoflavone
LysoPC(P-18:0)	HMDB13122	3.6	0.04	508.37841	30.2		Lipid
Indoxyl	HMDB04094	2.9	0.01	134.05988	16.0	Y	Tryptophan
4-Hydroxyphenylacetylglutamine	HMDB06061	2.4	0.009	282.09739	11.4	Y	Amino acids and derivatives
Stearic acid	HMDB00827	2.2	0.02	285.27934	35.8		Fatty Acids
Cytosine	HMDB00630	2.1	0.009	112.05043	12.8		Pyrimidine
L-Methionine	HMDB00696	1.9	0.01	150.05808	2.7		Amino acids
Hydroxyisobutyric acid	HMDB00336	1.8	0.01	105.05504	2.7		Organic acid
3-Indolepropionic acid	HMDB02302	1.8	0.05	190.08627	19.5	Y	Indoles
Tyramine	HMDB00306	1.8	0.04	138.09133	4.3	Y	Biogenic amine
N6-Acetyl-L-lysine	HMDB00206	1.7	0.02	189.12315	15.0		Amino acids and derivatives
Indole-3-carboxylic acid	HMDB03320	1.7	0.02	162.05471	15.1	Y	Indoles
Hippuric acid	HMDB00714	1.7	0.04	180.06588	15.1	Y	Amino acids and Derivatives
2-Phenylglycine	HMDB02210	1.6	0.05	152.07037	15.1		Amino acids and derivatives
Cuminaldehyde	HMDB02214	1.6	0.04	149.09581	16.7		
Propionylcarnitine	HMDB00824	1.6	0.009	218.13825	3.5		Acyl carnitines
Indoleacrylic acid	HMDB00734	1.5	0.03	188.07051	20.4	Y	Indoles
Salicylic acid	HMDB01895	1.5	0.01	139.03878	12.8		Benzoic acid and derivatives
N-Acetylvanilalanine	HMDB11716	1.5	0.01	254.10265	14.9		Amino acids and derivatives
4-(2-Aminophenyl)-2,4-dioxobutanoic acid	HMDB00978	1.5	0.0005	208.06045	14.8		Tryptophan metabolism
Salicyluric acid	HMDB00840	-1.5	0.004	196.06063	11.1		Amino acids and derivatives
N-Nonanoylglycine	HMDB13279	-1.5	0.0009	216.15943	25.7		Amino acids and derivatives
Octanoylcarnitine	HMDB00791	-1.5	0.04	288.2174	21.7		Acyl carnitines
Dodecanedioic acid	HMDB00623	-1.5	0.01	231.15882	21.6		Fatty acids
Dodecenoylcarnitine	HMDB13326	-1.5	0.007	342.26408	25.5		Acyl carnitines
9-Decenoylcarnitine	HMDB13205	-1.5	0.0005	314.23313	22.9		Acyl carnitines
Acetylcysteine	HMDB01890	-1.5	0.005	164.03729	25.1		Amino acids and derivatives
Hydroxyphenylacetic acid	HMDB00440	-1.5	0.03	153.05475	19.5	Y	Phenylalanine metabolism
2-Hexenoylcarnitine	HMDB13161	-1.6	0.005	258.16807	18.2		Acyl carnitines
Decanoylcarnitine	HMDB00651	-1.6	0.04	316.24841	24.2		Acyl carnitines
2,8-Dihydroxyquinoline-beta-D-glucuronide	HMDB11658	-1.6	0.02	338.08789	10.5	Y	Glucuronides

Name	HMDB ID[a]	Fold	*p* value	Mass	Retention time	Gut flora related	Pathways or functions
3-Indole carboxylic acid glucuronide	HMDB13189	-1.7	0.002	338.08819	4.3	Y	Glucuronides
Pyrrolidonecarboxylic acid	HMDB00805	-1.8	0.0006	130.04981	25.1		Glutamine metabolism
3-Dehydroxycarnitine	HMDB06831	-1.8	0.003	146.11625	2.6		Acyl carnitines
Benzaldehyde	HMDB06115	-1.9	0.03	107.04916	17.6	Y	
3-Methyldioxyindole	HMDB04186	-2.1	0.0006	164.07013	16.3	Y	Tryptophan metabolism
Phenylacetaldehyde	HMDB06236	-2.1	0.03	121.06421	13.4	Y	Phenylalanine metabolism
Coumarin	HMDB01218	-2.9	0.03	147.0439	15.8		
6-Hydroxy-5-methoxyindole glucuronide	HMDB10362	-3.0	0.002	340.10401	5.0	Y	Glucuronides
5-Methyltetrahydrofolic acid	HMDB01396	-9.9	0.03	460.19541	9.0		

[a]The metabolites were indexed using the IDs in the Human Metabolome Database (HMDB, http://www.hmdb.ca/).

Supplemental Material, Table S4. Identified significantly changed metabolites (fold change >1.5 $p < 0.05$, compared to controls) in plasma samples.

Name	HMDB ID[a]	Fold	p value	Mass	Retention Time (RT)	Gut flora related	Pathways
Phenylacetylglycine	HMDB00821	2.22	0.03	194.0805	16.9	Y	Amino acids and derivatives
LysoPC(20:4)	HMDB10395	1.53	0.02	544.3362	29.1		Lipids
Cytosine	HMDB00630	1.52	0.03	112.051	2.3		Pyrimidine metabolism
LysoPC(22:6)	HMDB10404	1.5	0.03	568.3351	28.1		Lipids
LysoPC(15:0)	HMDB10381	-1.5	0.009	482.3198	28.1		Lipids
Phenylpyruvic acid	HMDB00205	-1.5	0.05	165.0538	8.8	Y	Phenylalanine metabolism
Heptadecanoyl carnitine	HMDB06210	-1.62	0.04	414.354	27.2		Acyl carnitines
3-Methylglutarylcarnitine	HMDB00552	-1.64	0.02	290.1601	5.8		Acyl carnitines
3-Dehydroxycarnitine	HMDB06831	-1.75	0.03	146.117	2		Acyl carnitines
Lipoic acid	HMDB01451	-2.48	0.006	207.0512	1.7		Lipoic acid metabolism

[a]The metabolites were indexed using the IDs in the Human Metabolome Database (HMDB, http://www.hmdb.ca/).

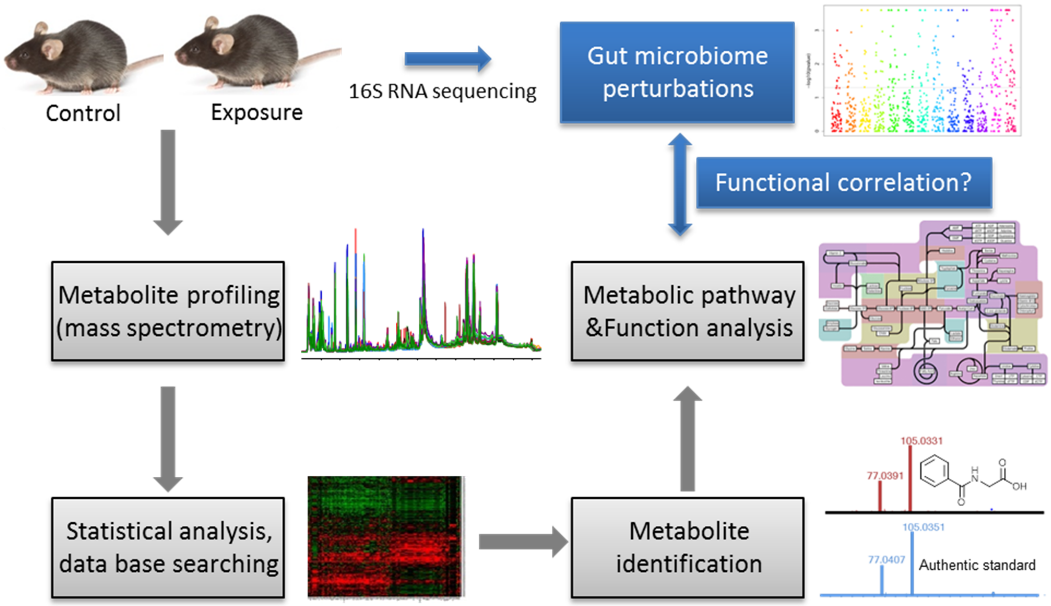

Supplemental Material, Figure S1. The integration approach combining 16S rRNA gene sequencing and mass spectrometry-based metabolomics profiling to explore the impact of arsenic exposure on the gut microbiome and its metabolomic profile.

A.

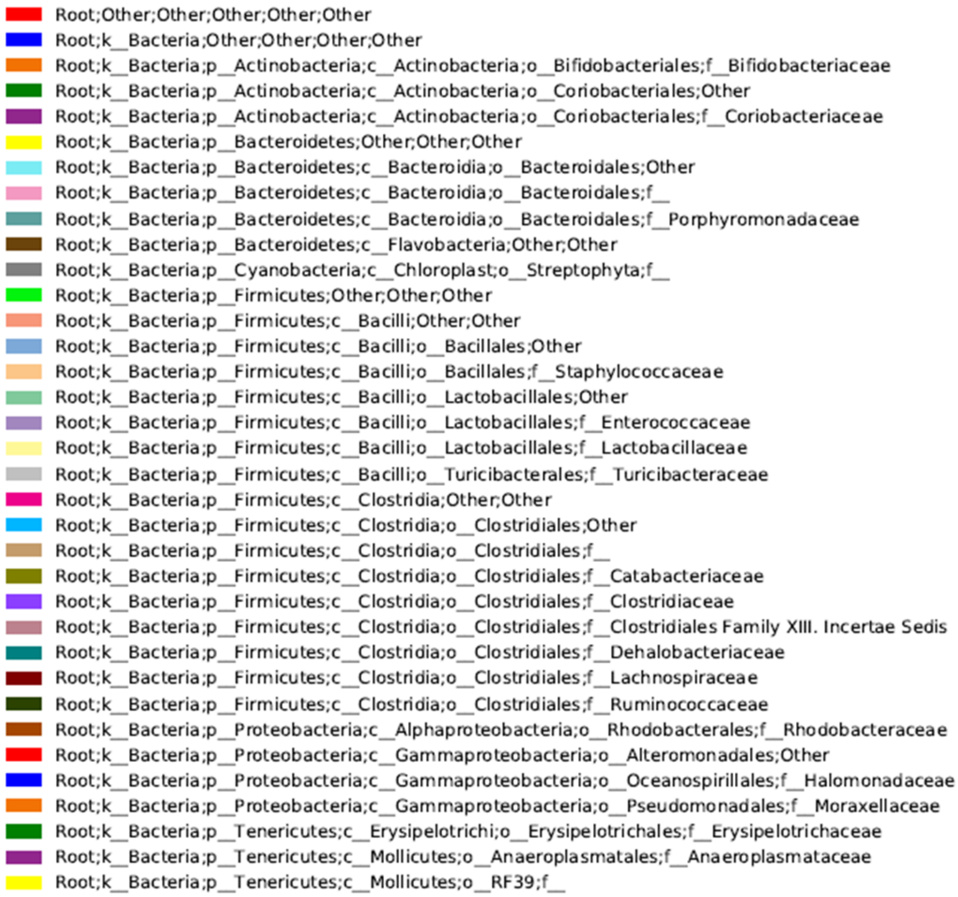

B.

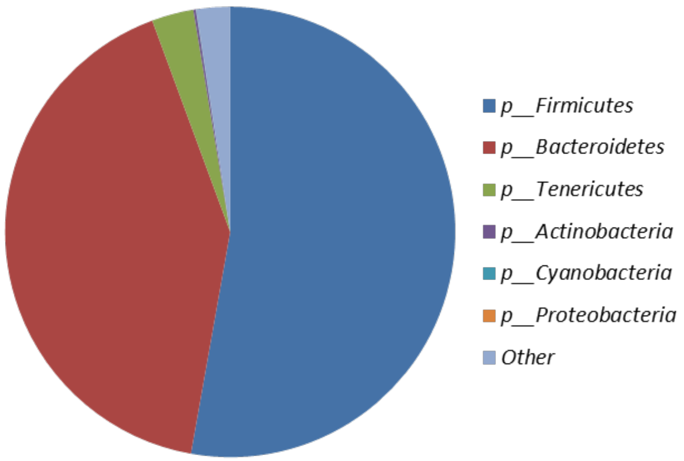

Supplemental Material, Figure S2. The taxonomic assignments of gut bacterial families, obtained using the Ribosomal Database Project classifier, with each color representing an individual bacterial family (A). Predominant gut bacteria at *phylum* level revealed by 16S rRNA sequencing, with *p_Firmicutes* and *p_Bacteroidetes* being the top 2 *phyla* (B).

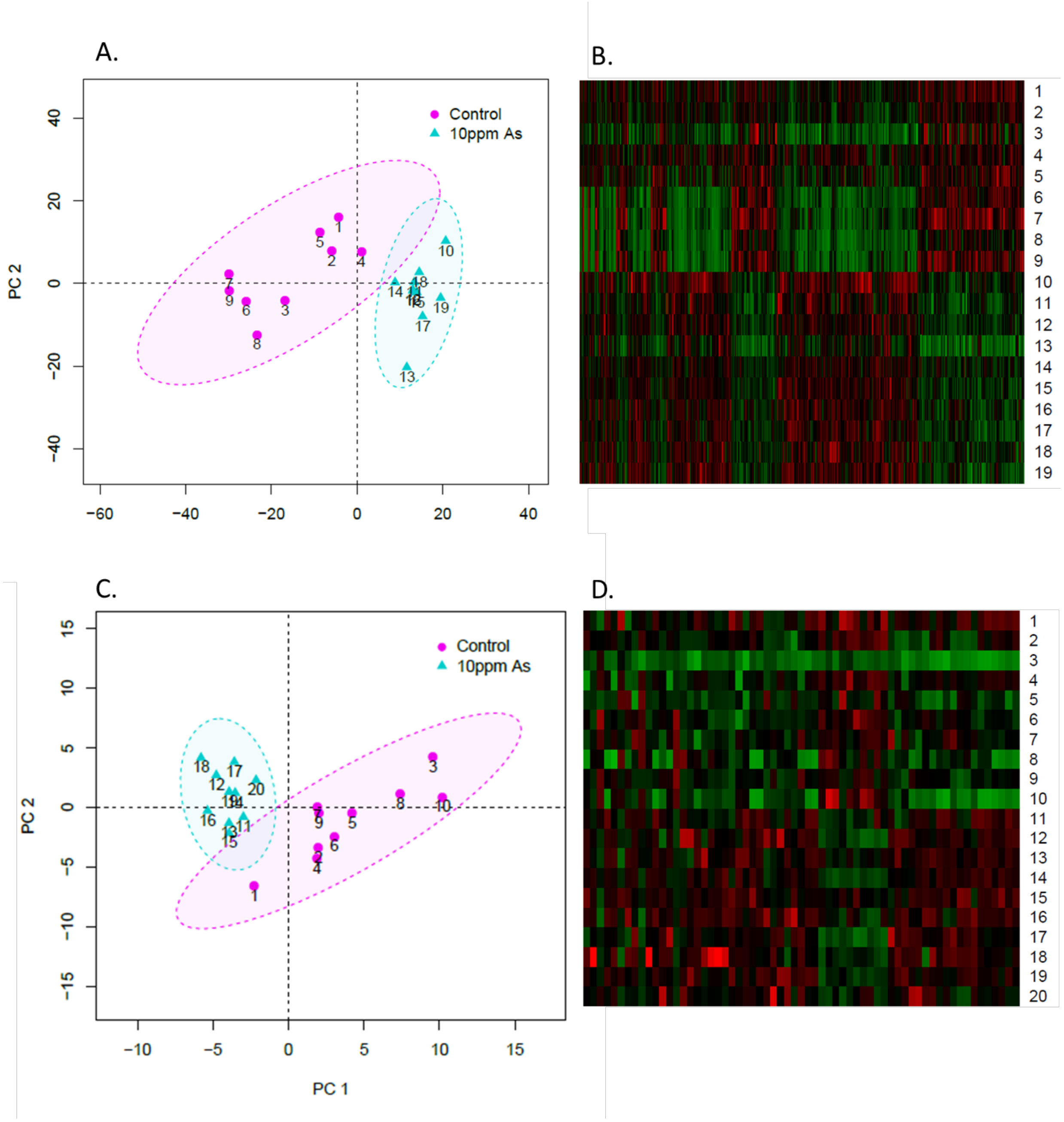

Supplemental Material, Figure S3. Separation of control and arsenic-treated mice using urine metabolite profiles by principal component analysis (A); The hierarchical clustering heat map constructed using urine molecular features with 1.5 fold changes ($p < 0.05$) shows a consistent clustering pattern within individual groups (B); Separation of control and arsenic-treated mice using metabolite profiles of plasma by principal component analysis (C); The hierarchical clustering heat map constructed using plasma molecular features with 1.5 fold changes ($p < 0.05$) (D).

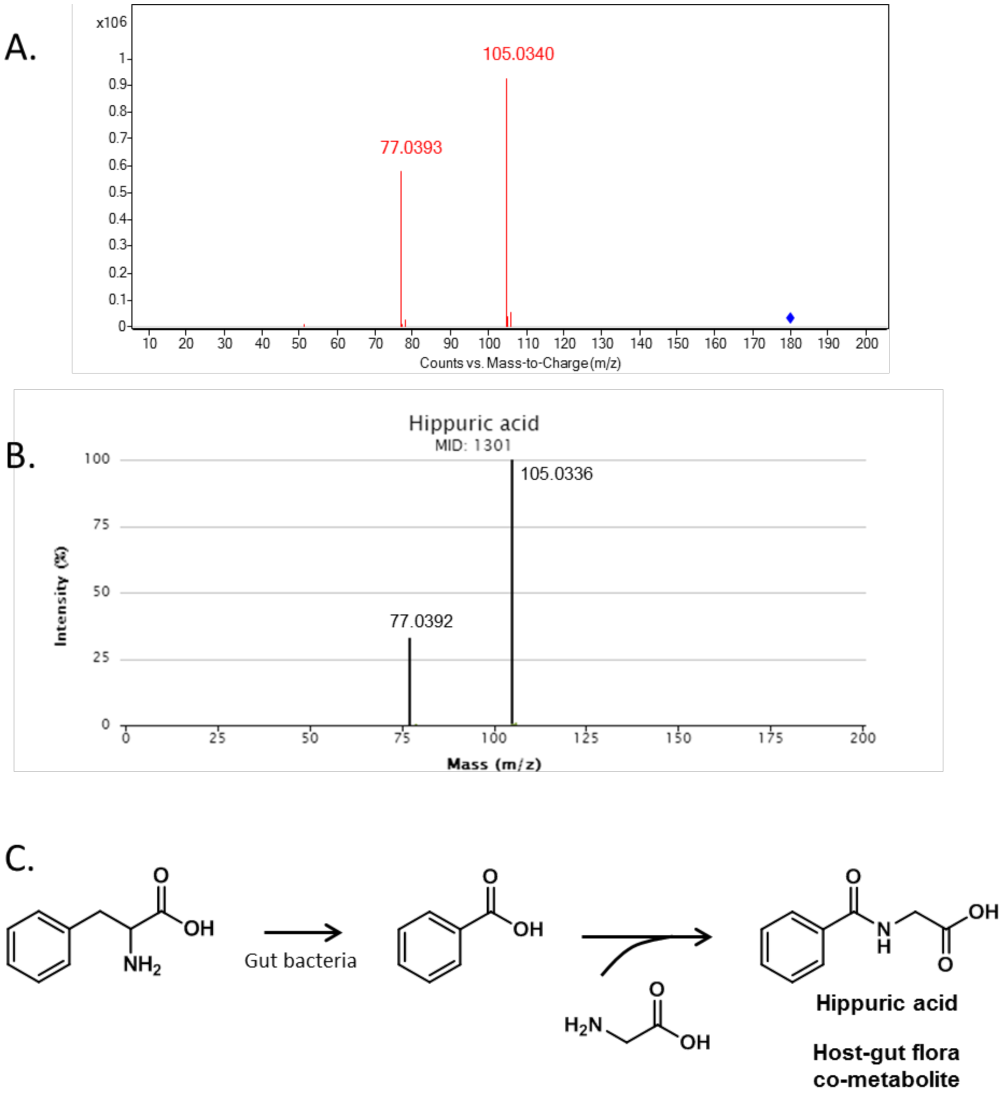

Supplemental Material, Figure S4. MS/MS spectrum of *m/z* =180.0658 at 15 min identifies this metabolite as hippuric acid (A: urine metabolite; B: the MS/MS spectrum of hippuric acid in the database); Hippuric acid is synthesized via phenylalanine metabolism and has been identified as a host-gut flora co-metabolite (C). (Database searching, using the exact mass at *m/z* 180.0658, gave 5-10 hits depending on the database. The product-ion spectrum arising from *m/z* 180.0658 at 15.1 min included characteristic hippuric acid fragments at *m/z* 77.0393 and *m/z* 105.0340, consistent with the fragmentation pattern in the database and for an authentic standard. Further metabolic pathway analysis illustrates that hippuric acid is a host-gut flora co-metabolite and its abundance is strongly associated with imbalanced gut microbiome composition).

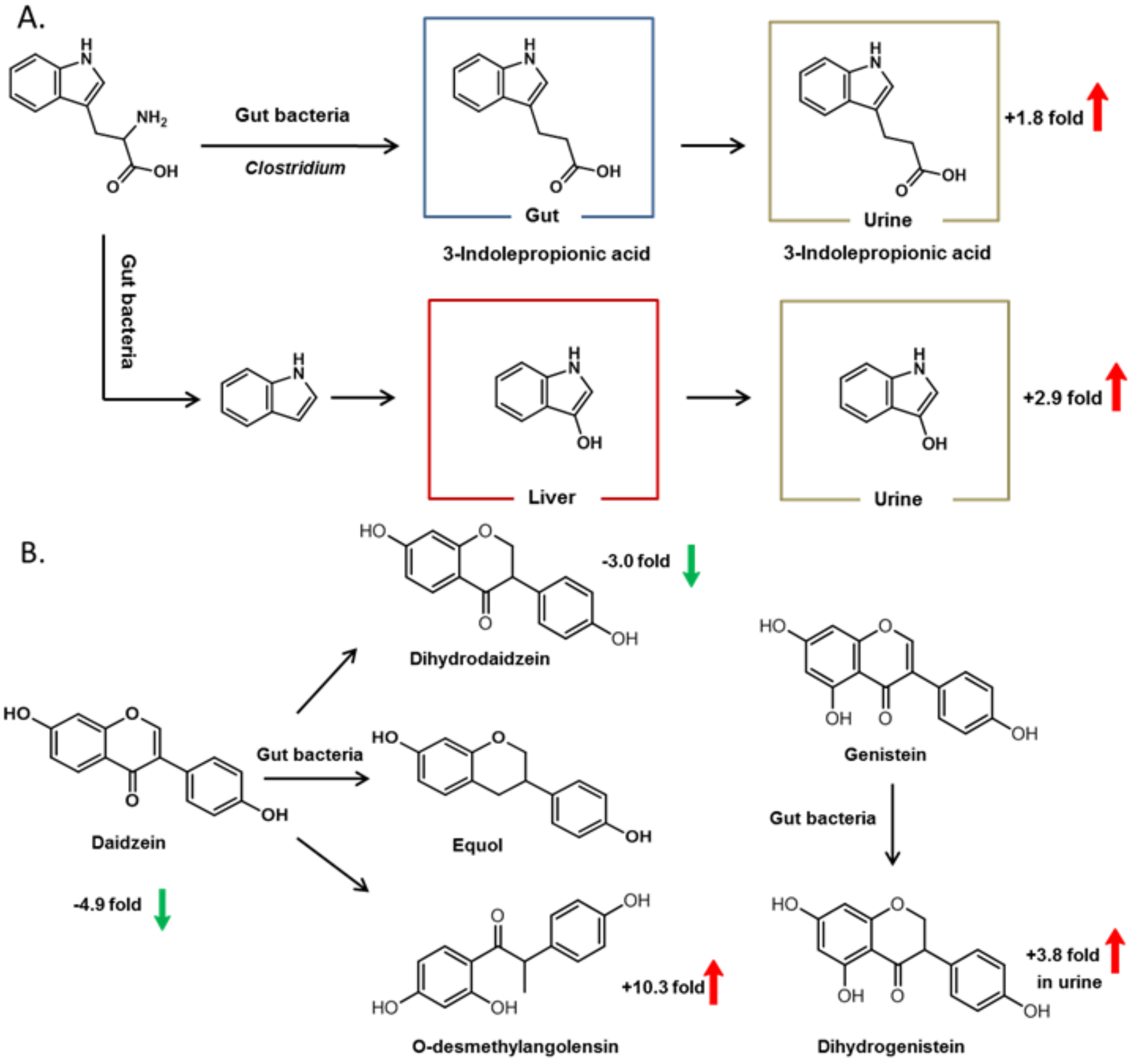

Supplemental Material, Figure S5. The influence of arsenic-induced gut microbiome perturbations on the formation of some typical gut-flora-generated fecal and urine metabolites. Indole derivatives were significantly regulated during exposure, with indolepropionic acid, indoxyl and others being increased in urine (A); Alterations of several isoflavone metabolites also clearly illustrate the effects of arsenic-induced gut flora changes, with daidzein and dihydrodaidzein being down-regulated, and O-desmethylangolensin and dihydrogenistein being up-regulated in feces and urine, respectively (B).
